# Clinical Outcomes for Patients With Monomicrobial vs Polymicrobial *Acinetobacter baumannii-calcoaceticus* Complex Infections Treated With Sulbactam-Durlobactam or Colistin: A Subset Analysis From a Phase 3 Clinical Trial

**DOI:** 10.1093/ofid/ofae140

**Published:** 2024-03-20

**Authors:** Sarah M McLeod, Alita A Miller, Khurram Rana, David Altarac, Samir H Moussa, Adam B Shapiro

**Affiliations:** Entasis Therapeutics, Inc, an affiliate of Innoviva Specialty Therapeutics, Inc, Waltham, Massachusetts, USA; Entasis Therapeutics, Inc, an affiliate of Innoviva Specialty Therapeutics, Inc, Waltham, Massachusetts, USA; Entasis Therapeutics, Inc, an affiliate of Innoviva Specialty Therapeutics, Inc, Waltham, Massachusetts, USA; Entasis Therapeutics, Inc, an affiliate of Innoviva Specialty Therapeutics, Inc, Waltham, Massachusetts, USA; Entasis Therapeutics, Inc, an affiliate of Innoviva Specialty Therapeutics, Inc, Waltham, Massachusetts, USA; Entasis Therapeutics, Inc, an affiliate of Innoviva Specialty Therapeutics, Inc, Waltham, Massachusetts, USA

**Keywords:** *Acinetobacter*, pneumonia, polymicrobial infection, sulbactam-durlobactam, carbapenem-resistant

## Abstract

**Background:**

In a previous study, the efficacy and safety of sulbactam-durlobactam vs colistin for the treatment of patients with carbapenem-resistant *Acinetobacter baumannii-calcoaceticus* complex (CRABC) infections were evaluated in a randomized controlled phase 3 trial. Both arms were dosed on a background of imipenem-cilastatin to treat coinfecting gram-negative pathogens. Thirty-six percent of infections in the primary efficacy population were polymicrobial.

**Methods:**

A subset analysis was performed to compare clinical and microbiological outcomes at test of cure (7 ± 2 days after the last dose) for patients with monomicrobial and polymicrobial CRABC infections. Minimal inhibitory concentrations of antibiotics against baseline isolates were determined by broth microdilution according to Clinical and Laboratory Standards Institute methodology.

**Results:**

Clinical cure, 28-day all-cause mortality, and microbiological outcomes were similar for patients in the sulbactam-durlobactam treatment arm with monomicrobial or polymicrobial *A baumannii-calcoaceticus* infections. Patients in the colistin arm with monomicrobial CRABC infections had higher mortality rates with worse clinical and microbiological outcomes as compared with those with polymicrobial infections. For patients who received sulbactam-durlobactam, imipenem susceptibility of coinfecting gram-negative pathogens trended with clinical benefit for patients with polymicrobial *A baumannii-calcoaceticus* infections. When tested in vitro, durlobactam restored imipenem susceptibility to the majority of coinfecting gram-negative pathogens from the sulbactam-durlobactam arm. This phenotype appeared to be related to the clinical outcome in 13 of 15 evaluable cases.

**Conclusions:**

These results suggest that the use of sulbactam-durlobactam plus a carbapenem could be an effective approach to treat polymicrobial infections that include CRABC, but additional clinical data are needed to demonstrate efficacy.

## Teaser

In subset analyses of phase 3 clinical trial data, patients who received sulbactam-durlobactam on a background therapy of imipenem-cilastatin had similar outcomes for monomicrobial or polymicrobial *Acinetobacter baumannii-calcoaceticus* complex infections.

Infections caused by carbapenem-resistant *Acinetobacter baumannii* are among the greatest challenges currently faced by physicians around the world treating gram-negative bacterial infections. This pathogen, the predominant representative of a larger collection of organisms referred to as the *Acinetobacter baumannii-calcoaceticus* complex (ABC), is notorious for its high rates of antibiotic resistance [1]. Clinical resistance to nearly all available antibiotic agents is widespread, with increasing rates of extensively drug-resistant and pandrug-resistant isolates [2]. Infections caused by *A baumannii* have serious consequences in health care settings due to increased cost, longer length of hospital stay, and high rates of morbidity and mortality [3].

Sulbactam-durlobactam is a pathogen-focused β-lactam/β-lactamase inhibitor combination with targeted antibacterial activity against ABC. As described by Kaye et al [4], sulbactam-durlobactam met criteria for noninferiority vs colistin for 28-day all-cause mortality (28D ACM) in patients with hospital-acquired bacterial pneumonia (HABP), ventilator-associated bacterial pneumonia (VABP), ventilated pneumonia, or bloodstream infections due to carbapenem-resistant ABC (CRABC) in a global phase 3 trial. Treatment with sulbactam-durlobactam was also associated with substantial improvements in clinical cure and microbiological eradication as compared with colistin. Sulbactam-durlobactam was well tolerated by this critically ill population, with a significantly lower incidence of nephrotoxicity vs colistin [4]. Sulbactam-durlobactam was approved in May 2023 by the US Food and Drug Administration for the treatment of HABP/VABP caused by susceptible ABC [5]. Additional supportive clinical evidence for the safety and efficacy of sulbactam-durlobactam has been reported for several compassionate use cases [6–9].

Polymicrobial HABP/VABP is frequently encountered in hospital settings [10]. Accordingly, patients in both treatment arms of the phase 3 trial also received a background of imipenem-cilastatin therapy [4]. This antibiotic was selected for background therapy as it was considered an effective agent for the treatment of not only carbapenem-susceptible ABC infections but also most potential coinfecting pathogens. In addition, imipenem-cilastatin and sulbactam-durlobactam have similar dosing regimens.

The following report is a secondary analysis of the sulbactam-durlobactam phase 3 trial that describes the incidence and outcomes of patients with polymicrobial vs monomicrobial CRABC infections, followed by an ad hoc in vitro analysis of the imipenem susceptibility of coinfecting gram-negative pathogens. The trial was not designed to measure the contribution of imipenem-cilastatin to efficacy against ABC in either arm of the trial or its efficacy against coinfecting pathogens. However, this retrospective analysis supports the choice of imipenem as background therapy for this pathogen-specific phase 3 trial and provides preliminary in vitro data to support the hypothesis that sulbactam-durlobactam plus a carbapenem may effectively treat polymicrobial infections that include ABC.

## METHODS

### Trial Design and Outcome Assessment

The trial design and methodology were fully described by Kaye et al [4]. Briefly, a phase 3 multinational noninferiority trial based on a randomized active-controlled design was conducted from September 2019 to July 2021 (ClinicalTrials.gov NCT03894046), and 59 clinical sites enrolled patients in 16 countries [4]. This trial had 2 parts. Part A was the assessor-blind and randomized portion that evaluated the efficacy and safety of sulbactam-durlobactam as compared with colistin in patients with documented ABC HABP, VABP, ventilated pneumonia, or bloodstream infection. Part B was an open-label observational study of sulbactam-durlobactam treatment for patients with ABC infections who did not tolerate colistin or whose ABC pathogens were resistant to colistin/polymyxin B. Imipenem-cilastatin was used as background therapy in all treatment groups. Concomitant therapy with any additional or adjunctive non–study-specific gram-negative antibiotic was not allowed. Clinical outcome assessments, including whether cause of death was associated with the index infection, were performed by a blinded assessor. Any organism isolated from the blood or infection site–specific cultures was identified to genus and species by the local laboratory. Microbiological outcome assessments were based on the presence or absence of ABC in cultures from each patient. Organisms were cultured and quantified at the local laboratory, and susceptibility of the organisms was performed per local laboratory standards. Bacterial isolates cultured at the local laboratory were sent to the central laboratory for confirmation of identification and susceptibility testing. A small subset of clinical sites was unable to ship isolates due to the COVID-19 pandemic. In these cases, species determination and carbapenem susceptibility data generated at the local laboratory were used in the analysis [4].

Patients were considered to have a gram-negative polymicrobial ABC infection if at least 1 other gram-negative pathogen in addition to ABC was isolated at screening [4]. A favorable microbiological assessment included ABC infections that were documented to be eradicated by the local laboratory or presumed eradicated due to clinical cure. Presumed persistence or eradication refers to cases where no microbiological sample was taken and the clinical outcome was failure or cure, respectively. Because the study was a pathogen-specific trial, the microbiological assessment documented only ABC eradication or persistence and did not include an assessment of coinfecting pathogens.

### Antibiotic Susceptibility Testing and Whole Genome Sequencing

Frozen bacterial cultures were sent to the central laboratory (IHMA, Inc). Isolates were subcultured and speciation performed by MALDI-TOF mass spectrometry. Minimum inhibitory concentrations (MICs) were determined by broth microdilution following Clinical and Laboratory Standards Institute guidelines [11]. Two testing paradigms were utilized to evaluate the ability of durlobactam, sulbactam, or sulbactam-durlobactam to restore imipenem or meropenem susceptibility to imipenem-nonsusceptible coinfecting non-ABC gram-negative pathogens. For Enterobacterales (because durlobactam alone has antibacterial activity against some species of this order), the following combinations were titrated in 2-fold dilutions: sulbactam-durlobactam, carbapenem-sulbactam, and carbapenem-durlobactam in 1:1 ratios and carbapenem-sulbactam-durlobactam in 1:1:1 ratios. For nonfermenting organisms, imipenem or meropenem alone or in a 1:1 ratio with sulbactam was titrated in 2-fold dilutions in the presence of 4 µg/mL of durlobactam. Two imipenem-resistant *Klebsiella pneumoniae* isolates that were also resistant to imipenem in the presence of durlobactam were subjected to whole genome sequencing analysis by methods previously described [12]. Amino acid sequences of genes of interest were compared with reference strain KPNIH1 (GenBank accession CP008827.1). The β-lactamase content of each strain was determined by BLAST within the CLC Genomics Workbench version 22.0 against an assembled database of genes curated at Entasis Therapeutics, Inc, with sequences originating from the NCBI Bacterial Antimicrobial Resistance Reference Gene Database (accession PRJNA313047).

### Patient Consent Statement

Each patient or legally authorized representative provided written informed consent before study participation as previously described [4].

## RESULTS

### Patient Demographics

As described by Kaye et al [4], patients enrolled in the microbiologically modified intent-to-treat population (m-MITT) were those who received any amount of study drug and had an ABC organism isolated at baseline. Of these, 128 patients were in the part A CRABC m-MITT primary efficacy population (m-MITT patients with carbapenem-resistant ABC that were also susceptible to the study drug), with equal numbers of patients (n = 64) receiving sulbactam-durlobactam or colistin, each on a background of imipenem-cilastatin [4]. Most of these patients had HABP (n = 55) or VABP (n = 68); 3 additional patients had ventilated pneumonia and 3 had bacteremia [4]. Thirty-six percent of all baseline infections in the CRABC m-MITT population were polymicrobial (Table 1), with a higher incidence observed in the sulbactam-durlobactam arm (27/64, 42%) than the colistin arm (19/64, 30%) [4]. In the open-label part B portion of the study, 23 of 28 (82%) patients had monomicrobial ABC infections. The majority (57%, 16/28) of part B patients had bacteremia; 8 had VABP and 4 had HABP [4]. In secondary analyses of the monomicrobial and polymicrobial CRABC populations, the proportion of monomicrobial infections was consistently higher than polymicrobial infections across regions (Table 1).

**Table 1.** Monomicrobial vs Polymicrobial *Acinetobacter baumannii-calcoaceticus* Complex Infections at Baseline by Treatment Arm and Region: Part A of the CRABC m-MITT Population

		Treatment Arm		Region				
Category	Total	SUL-DUR	COL	North America	Europe	Latin America	Asia Pacific	China
Total	128	64	64	1	53	17	23	34
Monomicrobial	82 (64)	37 (58)	45 (70)	0 (0)	32 (61)	10 (59)	15 (65)	25 (74)
Polymicrobial	46 (36)	27 (42)	19 (30)	1 (100)	21 (39)	7 (41)	8 (35)	9 (26)

Data are presented as No. (%). The CRABC m-MITT population consisted of those who received any amount of study drug and had a CRABC organism isolated at baseline.

Abbreviations: COL, colistin; CRABC, carbapenem-resistant *Acinetobacter baumannii-calcoaceticus*; m-MITT, microbiologically modified intent to treat; SUL-DUR, sulbactam-durlobactam.

### Types of Polymicrobial Gram-Negative ABC Infections

Most patients in part A (85%, 39/46) with polymicrobial ABC infections were infected with ABC plus 1 other gram-negative pathogen. Ten patients had 2 and 1 patient had 3 coinfecting gram-negative pathogens. The most common coinfecting gram-negative pathogen was *Klebsiella pneumoniae*, followed by *Pseudomonas aeruginosa*, both of which demonstrated high rates of imipenem nonsusceptibility (77% [17/22] and 58% [7/12], respectively). Four patients were coinfected with *Stenotrophomonas maltophilia*, which is intrinsically carbapenem resistant, and 5 patients were coinfected with *Proteus mirabilis*, which is frequently imipenem-nonsusceptible due to non–β-lactamase-mediated mechanisms. Other coinfecting gram-negative pathogens isolated from these patients included *Achromobacter xylosoxidans*, *Escherichia coli*, *Klebsiella oxytoca*, and *Serratia marcescens*, all of which were imipenem susceptible. Overall, 57% (32/56) of coinfecting gram-negative pathogens from both arms of part A were nonsusceptible to imipenem (19/33 in the sulbactam-durlobactam arm, 13/23 in the colistin arm; Supplementary Tables 1 and 2). In part B, 4 of the 5 patients with polymicrobial ABC infections included gram-negative organisms, 3 of whom had organisms that were imipenem nonsusceptible.

### Outcomes for Monomicrobial vs Polymicrobial ABC Infections

Three patients in part A withdrew consent before having survival status assessed at day 28 and were not included in the primary efficacy analysis [4]. Therefore, efficacy analysis was performed on 63 patients in the sulbactam-durlobactam arm and 62 patients in the colistin arm. This population was defined as the CRABC m-MITT. The overall 28D ACM for patients in the CRABC m-MITT population in part A was numerically higher in the colistin arm (32.3%, 20/62) than in the sulbactam-durlobactam arm (19%, 12/63). Similar trends favoring sulbactam-durlobactam were observed for clinical cure and favorable microbiological outcome at test of cure (TOC; 7 ± 2 days after last dose) [4]. In this study, 28D ACM, clinical cure, and microbiological outcomes for monomicrobial and polymicrobial outcomes were compared for both treatment arms. The 28D ACM for patients in the CRABC m-MITT population in part A with monomicrobial or polymicrobial CRABC infections is shown in Table 2. The rate of 28D ACM in the sulbactam-durlobactam arm was similar between patients with monomicrobial infections (16.7%, 6/36) and polymicrobial infections (22.2%, 6/27). The rate of 28D ACM in the colistin arm was higher in patients with monomicrobial infections (34.9%, 15/43) than in those with polymicrobial infections (26.3%, 5/19).

**Table 2.** Clinical Outcomes for the CRABC m-MITT Primary Efficacy Population for Monomicrobial vs Polymicrobial ABC Infections

	Part A		Part B
Type of Infection	SUL-DUR + IMI	COL + IMI	SUL-DUR + IMI
All ABC infections	63	62	28
28-d all-cause mortality	12 (19)	20 (32.3)	5 (17.9)
Clinical cure at TOC	39 (61.9)	25 (40.3)	20 (71.4­)
Favorable microbiological assessment^a^ at TOC	43 (68.2)	26 (41.9)	22 (78.6)
Monomicrobial ABC infections	36	43	23
28-d all-cause mortality	6 (16.7)	15 (34.9)	3 (13)
Clinical cure at TOC	23 (63.9)	15 (34.9)	17 (73.9­)
Favorable microbiological assessment^a^ at TOC	24 (66.7)	14 (32.6)	18 (78.3)
All polymicrobial ABC infections	27	19	5
28-d all-cause mortality	6 (22.2)	5 (26.3)	2 (40)
Clinical cure at TOC	16 (59.3)	10 (52.6)	3 (60)
Favorable microbiological assessment^a^ at TOC	19 (70.4)	12 (63.2)	4 (80)
Gram-negative polymicrobial ABC infections	26	18	4
IPM susceptible only: non-ABC	11	5	1
28-d all-cause mortality	1 (9.1)	1 (20)	1 (100)
Clinical cure at TOC	5 (45)	4 (80)	0 (0)
IPM nonsusceptible: non-ABC	15	13	3
28-d all-cause mortality	4 (26.7)	3 (23.1)	1 (33)
Clinical cure at TOC	10 (66.7)	6 (46.2)	2 (67)

Data are presented as No. (%). Data exclude patients who had withdrawn consent or were lost to follow-up. The CRABC m-MITT population consisted of those who received any amount of study drug and had a carbapenem-resistant ABC organism isolated at baseline.

Abbreviations: ABC, *Acinetobacter baumannii-calcoaceticus* complex; COL, colistin; IMI, imipenem-cilastatin; IPM, imipenem; m-MITT, microbiologically modified intent to treat; SUL-DUR, sulbactam-durlobactam; TOC, test of cure.

^a^Eradication or presumed eradication of baseline ABC infection.

As shown in Table 3, of the 12 patients in the sulbactam-durlobactam arm who did not survive to 28 days, 4 deaths (33.3%) were attributed to the index infection (2/6 [33.3%] for monomicrobial and polymicrobial ABC infections). Of the 20 patients in the colistin arm who did not survive to 28 days, 10 (50%) were attributed to the index infection: 7 of 15 (46.7%) with monomicrobial ABC infections and 3 of 5 (60%) with polymicrobial ABC infections.

**Table 3.** Deaths Attributable to Baseline Index Infection by Treatment Arm and Monomicrobial vs Polymicrobial ABC Infection: Part A of the Carbapenem-Resistant ABC m-MITT Population

	Deaths, No. (%)^a^							
	Sulbactam-Durlobactam				Colistin			
Category	Total	ABC Infection	ABC + Coinfection	Death Due to Baseline Infection	Total	ABC Infection	ABC + Coinfection	Death Due to Baseline Infection
All	12	2 (16.6)	2 (16.6)	4 (33)	20	7 (35)	3 (15)	10 (50)
Monomicrobial	6	2	NA	2 (33)	15	7	NA	7 (46.7)
Polymicrobial	6	0	2 ^b^	2 (33)	5	0	3 ^c^	3 (60)

The CRABC m-MITT population consisted of those who received any amount of study drug and had a carbapenem-resistant ABC organism isolated at baseline.

Abbreviations: ABC, *Acinetobacter baumannii-calcoaceticus* complex; m-MITT, microbiologically modified intent to treat; NA, not applicable.

^a^Both arms were dosed on a background of imipenem-cilastatin.

^b^Both patients had imipenem-resistant coinfecting pathogens.

^c^One of these was documented by the treating physician as being caused by the index infection; the other 2 were added to this category for the purposes of this evaluation because they died of pneumonia and multiorgan failure with persistent ABC and colistin- and imipenem-resistant coinfection.

Similar trends were observed for clinical cure and microbiological outcomes. For patients in the sulbactam-durlobactam arm, the rate of clinical cure at TOC was 63.9% (23/36) for monomicrobial infections and 59.3% (16/27) for polymicrobial infections. In contrast, for patients who received colistin, clinical cure at TOC was 34.9% (15/43) for monomicrobial infections and 52.6% (10/19) for polymicrobial infections. Likewise, the percentage of favorable microbiological outcomes at TOC for patients in the sulbactam-durlobactam arm was 66.7% (24/36) for monomicrobial infections and 70.4% (19/27) for polymicrobial infections. For patients in the colistin arm, the percentage of favorable microbiological outcomes at TOC was 32.6% (14/43) for monomicrobial ABC infections and 63.2% (12/19) for polymicrobial infections (Table 2).

Taken together, these results demonstrate that patients in part A who received sulbactam-durlobactam on a background of imipenem-cilastatin had similar outcomes for monomicrobial or polymicrobial infections and had numerically greater clinical cure and microbiological eradication rates than those treated with colistin plus imipenem-cilastatin. In contrast, patients in the colistin arm with monomicrobial ABC infections had higher mortality rates with lower clinical cure and microbiological eradication rates as compared with those with polymicrobial ABC infections.

For part B, the 28D ACM was 17.9% (5/28 evaluable patients) [4]. Those who did not survive included 13% (3/23) of patients with monomicrobial ABC infections and 40% (2/5) with polymicrobial ABC infections (Table 2). The rate of clinical cure at TOC in part B was 73.9% (17/23) for monomicrobial ABC infections and 60.0% (3/5) for polymicrobial ABC infections. The rate of microbiological favorable assessment at TOC in part B was 78.3% (18/23) for monomicrobial ABC infections and 80% (4/5) for polymicrobial ABC infections (Table 2).

### Imipenem Susceptibility of Coinfecting Gram-Negative Pathogens

Imipenem-cilastatin was used as background therapy in all treatment groups as it was predicted to be an effective therapy for common coinfecting pathogens [4]. In a retrospective analysis, the imipenem susceptibility of coinfecting pathogens was compared with clinical outcomes for patients with polymicrobial ABC infections. For this analysis, any polymicrobial ABC infections that did not contain non-ABC gram-negative organisms were excluded (n = 1 for both arms in part A and n = 1 for part B). In addition, microbiological outcomes were not considered because only ABC eradication or persistence was documented for this pathogen-specific trial.

A numerically higher percentage of part A patients in the sulbactam-durlobactam arm had imipenem-susceptible gram-negative coinfections than in the colistin arm (17.5% [11/63] vs 8.1% [5/62]; Table 2). A numerically similar percentage of patients in each arm had at least 1 baseline imipenem-nonsusceptible, gram-negative coinfection: 23.8% (15/63) in the sulbactam-durlobactam arm and 21.0% (13/62) in the colistin arm (Supplementary Tables 1 and 2).

### Imipenem Susceptibility of Gram-Negative Coinfecting Pathogens and Clinical Outcomes for Patients With Polymicrobial ABC Infections

Only 1 patient of 11 (9%) in the sulbactam-durlobactam arm and 1 of 5 (20%) in the colistin arm with an imipenem-susceptible gram-negative coinfection died by day 28. Neither death was considered related to the index infection. In this subgroup, a lower percentage experienced clinical cure at TOC in the sulbactam-durlobactam arm than in the colistin arm (5/11 [45%] vs 4/5 [80%]; Table 2).

Similar percentages of patients with imipenem-nonsusceptible gram-negative coinfections died by day 28 in the sulbactam-durlobactam arm (4/15 [26.7%]) vs the colistin arm (3/13 [23.1%]; Table 2). In this subgroup, 10 of 15 (67%) and 6 of 13 (46%) patients experienced clinical cure at TOC in the sulbactam-durlobactam and colistin arms, respectively. Two deaths in the sulbactam-durlobactam arm and 3 deaths in the colistin arm in this subgroup were considered related to the index infection (Tables 3, Supplementary Tables 1 and 2).

Taken together, these data suggest that patients in both treatment arms with gram-negative polymicrobial ABC infections experienced improved clinical outcomes when the coinfecting pathogen was imipenem susceptible. Nevertheless, many patients with imipenem-nonsusceptible gram-negative coinfections survived with favorable clinical outcomes in both treatment arms. For those patients in the colistin arm, this observation is likely to be due to the broad-spectrum gram-negative activity of colistin. Indeed, susceptibility testing of available isolates showed that all but 2 of the coinfecting gram-negative polymicrobial ABC infections in this arm were susceptible to either colistin (defined as colistin MIC ≤2 µg/mL for the purposes of this study) or imipenem (Supplementary Table 2). In contrast, the antibacterial activity of sulbactam is limited to a very small number of bacterial species, including ABC. However, durlobactam alone is a broad-spectrum inhibitor of serine β-lactamases that has been shown to restore carbapenem activity in vitro against carbapenem-resistant Enterobacterales and *P aeruginosa* [13]. It is therefore possible that some imipenem-nonsusceptible gram-negative coinfections in the sulbactam-durlobactam arm of the trial may have been restored to imipenem susceptibility by durlobactam.

### Restoration of Imipenem Susceptibility of Gram-Negative Coinfecting Pathogens by Durlobactam In Vitro

All available coinfecting, imipenem-nonsusceptible, gram-negative isolates from patients treated with sulbactam-durlobactam in parts A and B (n = 20 isolates from 16 patients; Supplementary Table 3) were tested for restoration of imipenem susceptibility in the presence of durlobactam alone, sulbactam alone, or the combination. Predictably, the 2 *S maltophilia* isolates available for testing remained imipenem resistant under all conditions tested, as *S maltophilia* encodes for metallo-β-lactamases that durlobactam does not inhibit. Imipenem susceptibility of the 3 *P mirabilis* isolates, which tested as intermediate (MIC, 2 µg/mL), was also unchanged by the addition of durlobactam, sulbactam, or the combination. In contrast, all 3 imipenem-resistant *P aeruginosa* isolates were rendered imipenem susceptible in the presence of durlobactam. Of the 12 imipenem-resistant *K pneumoniae* isolates, 8 (66%) became susceptible and 2 demonstrated intermediate susceptibility (17%) when durlobactam was added. Addition of sulbactam to imipenem alone or imipenem-durlobactam had little to no effect on overall susceptibility. Whole genome sequencing of the 2 imipenem-durlobactam–nonsusceptible *K pneumoniae* isolates revealed that the isolate from patient SUD-P18 encodes *bla*_NDM-1_, which durlobactam does not inhibit, and the isolate from patient SUD-P25 encodes frameshift mutations in several OmpK porins.

The in vitro susceptibilities of these 20 isolates to meropenem with and without durlobactam, sulbactam, or sulbactam-durlobactam were tested. The same patterns of restoration of carbapenem activity by durlobactam, but not sulbactam, were observed for meropenem as for imipenem, with the exception of the 3 *P mirabilis* isolates, all of which were susceptible to meropenem alone (Supplementary Table 3). These results suggest that meropenem could also be considered a background therapy for sulbactam-durlobactam against polymicrobial CRABC infections that include gram-negative coinfecting pathogens, depending on the species and antibiotic susceptibility of the pathogen.

### Imipenem-Durlobactam Susceptibility of Gram-Negative Coinfecting Pathogens and Clinical Outcomes for Patients With Polymicrobial ABC Infections

The clinical outcomes for patients who received sulbactam-durlobactam and had coinfecting gram-negative pathogens that were nonsusceptible to imipenem are summarized in Table 4. Durlobactam restored imipenem to susceptible or intermediate susceptibility criteria against coinfecting gram-negative pathogens of 9 patients, 8 of whom experienced favorable clinical outcomes. Of the 5 patients whose coinfections remained imipenem nonsusceptible in the presence of durlobactam, 3 did not survive to day 28 and 1 was considered a clinical failure at TOC. Three of these patients were infected with organisms with intrinsic imipenem resistance: patients SUD-P7 and SUD-P26 (both coinfected by *S maltophilia*) as well as SUD-P11 (coinfected by *P mirabilis*; Supplementary Table 3).

**Table 4.** IPM-DUR Susceptibility of Coinfecting Gram-Negative Pathogens and Clinical Outcomes in 15 Evaluable Patients With IPM-NS Polymicrobial ABC Infections Treated With SUL-DUR

Patient ID (Deidentified)	Day 28 Survival (Day of Death)	Clinical Outcome at TOC	Baseline Gram-Negative Coinfection: IPM Susceptibility	IPM Susceptibility Restored by DUR In Vitro?^b^	Correlation Between Clinical Outcome and IPM-DUR Activity In Vitro?^b^
SUD-P1	Alive	Cure	*P mirabilis*: Intermediate	No	Yes
			*K pneumoniae*: NS	Intermediate	
SUD-P7	Dead (19)	Cure	*S maltophilia*: NS	No	Yes
SUD-P11	Alive	Failure	*P mirabilis*: NS	No	Yes
SUD-P16	Alive	Indeterminant	*P aeruginosa*: NS	Yes	Yes
			*K pneumoniae*: NS	Intermediate	
SUD-P18	Alive	Cure	*K pneumoniae*: NS	No	No
SUD-P19^a^	Dead (17)	Failure	*K pneumoniae*: NS	Yes	No
SUD-P20	Alive	Cure	*K pneumoniae*: NS	Yes	Yes
SUD-P21	Alive	Cure	*K pneumoniae*: NS	Yes	Yes
			*P aeruginosa*: NS	Yes	
SUD-P22	Alive	Cure	*K pneumoniae*: NS	Yes	Yes
SUD-P23	Alive	Cure	*K pneumoniae*: NS	Yes	Yes
SUD-P24	Alive	Cure	*K pneumoniae*: NS	Yes	Yes
SUD-P25	Dead (5)	Failure	*K pneumoniae*: NS	No	Yes
SUD-P26^a^	Dead (20)	Failure	*S maltophilia*: NS	No	Yes
			*P aeruginosa*: Susceptible^c^	NA	
SUD-P29^d^	Alive	Cure	*K pneumoniae*: NS	Yes	Yes
SUD-P30^d^	Alive	Cure	*K pneumoniae*: NS	Yes	Yes

Evaluable patients were those whose infecting pathogens were sent to the central laboratory for in vitro susceptibility testing.

Abbreviations: ABC, *Acinetobacter baumannii-calcoaceticus* complex; DUR, durlobactam; IPM, imipenem; NA, not available; NS, nonsusceptible; SUL-DUR, sulbactam-durlobactam; TOC, test of cure.

^a^Death related to index infection.

^b^ *Yes* was defined as (1) patients who were alive at day 28, had a clinical cure at TOC, and had a baseline non-ABC gram-negative IPM-resistant isolate that was restored to susceptible in vitro in the presence of DUR or (2) patients who died by day 28 or had a clinical failure at TOC and the baseline isolate was not restored to IPM susceptibility in the presence of DUR. *No* was defined as (1) patients who were alive at day 28 and had a clinical cure at TOC but the baseline isolate was not restored to IPM susceptible in the presence of DUR or (2) patients who were not alive at day 28 or had a clinical failure at TOC but the baseline isolate was restored to susceptibility in the presence of DUR.

^c^Susceptible: minimum inhibitory concentration ≤1 µg/mL for Enterobacterales and ≤2 µg/mL for nonfermenters.

^d^Patients from part B.

Overall, the in vitro activity of the imipenem-durlobactam combination against coinfecting gram-negative pathogens was aligned with the clinical outcome in 13 of the 15 evaluable patients. The 2 patients with discordant results were SUD-P18 and SUD-P19. As mentioned previously, patient SUD-P18 had a coinfecting metallo-β-lactamase–encoding *K pneumoniae* that remained imipenem resistant in the presence of durlobactam. However, this patient survived to day 28 with a favorable clinical outcome and a fully eradicated CRABC infection at TOC. In contrast, despite in vitro evidence that durlobactam fully restored the in vitro imipenem susceptibility of the coinfecting *K pneumoniae*, patient SUD-P19 died from the index infection on day 17, despite showing eradication of the *A baumannii* after the end of sulbactam-durlobactam therapy on day 11.

## DISCUSSION

In a phase 3 trial, sulbactam-durlobactam met the primary efficacy outcome of noninferiority vs colistin 28D ACM in HABP/VABP cases with documented CRABC infections [4]. Both treatment arms were dosed with imipenem-cilastatin to treat any possible coinfecting gram-negative pathogens. The study detailed here presents a subset analysis from this phase 3 trial, comparing the clinical outcomes for patients with monomicrobial CRABC infections against those with polymicrobial CRABC infections.

It is important to note that the small number of evaluable patients is a significant limitation for subset analyses of the phase 3 trial. Nevertheless, in the case of a pathogen-targeted therapy such as sulbactam-durlobactam, it is important to examine if the clinical outcomes were influenced by the presence of non-CRABC coinfecting pathogens. Another limitation is that the phase 3 trial tracked just the microbial eradication of the CRABC isolates, so conclusions about efficacy against the non-CRABC pathogens can only be inferred from 28D ACM and clinical cure assessments. Also, while all patients had CRABC isolated at baseline as well as other signs and symptoms of infection [4], in the case of the polymicrobial infections, it is not possible to know which pathogens, if any, were asymptomatic colonizers.

This study demonstrated that patients in the colistin arm with monomicrobial CRABC infections had higher mortality rates with worse clinical and microbiological outcomes than those with polymicrobial ABC infections. Similarly favorable results were observed for patients who received sulbactam-durlobactam therapy with monomicrobial or polymicrobial CRABC infections, supporting the selection of imipenem-cilastatin as background therapy.

A number of patients with imipenem-nonsusceptible, coinfecting, gram-negative infections demonstrated favorable outcomes following administration of sulbactam-durlobactam and imipenem-cilastatin. Since durlobactam is a serine β-lactamase inhibitor that is capable of protecting carbapenems from hydrolysis by class A and D carbapenemases such as KPC and OXA-48 in non-ABC gram-negative organisms [13], it was considered possible that durlobactam restored imipenem activity against these imipenem-nonsusceptible coinfecting pathogens in the phase 3 trial. Therefore, a retrospective analysis was performed examining whether durlobactam restored imipenem activity in vitro and the clinical outcome of those patients. Although this was feasible for only 15 patients, an association between the ability of durlobactam to restore imipenem activity in vitro and outcomes for patients with mixed infections was observed for 13 (87%) of them. In vitro susceptibility test results also support consideration of meropenem as an effective background therapy for sulbactam-durlobactam against polymicrobial ABC infections, as in vitro results with meropenem-durlobactam combinations were similar to those observed with imipenem-durlobactam. However, the optimal background therapy with sulbactam-durlobactam for polymicrobial CRABC infections should be chosen according to the characteristics of the coinfecting pathogens. Taken together, these preliminary results support the generation of additional preclinical in vivo efficacy data and clinical data to explore the activity of durlobactam plus a carbapenem against carbapenem-resistant *P aeruginosa* and *K pneumoniae* pathogens.

## Supplementary Material

ofae140_Supplementary_Data

## Supplementary Data

Supplementary materials are available at *Open Forum Infectious Diseases* online. Consisting of data provided by the authors to benefit the reader, the posted materials are not copyedited and are the sole responsibility of the authors, so questions or comments should be addressed to the corresponding author.

## Notes

***Acknowledgments.*** We extend thanks to all the patients and their families, the investigators and study site personnel who took part in the clinical trial, and the personnel at IHMA, which served as the central laboratory.

***Data availability.*** All genome sequencing data described in this article have been deposited at DNA Data Bank of Japan/European Nucleotide Archive/GenBank as part of Bioproject PRJNA1073015, under the accession numbers JAZHFM000000000 and JAZHFN000000000. The versions described in this article are versions JAZHFM010000000 and JAZHFN010000000.

***Financial support*.** This work was supported by Entasis Therapeutics, Inc, an affiliate of Innoviva Specialty Therapeutics, Inc; and Zai Labs.
