# Supplementary material for: Clinical Outcomes for Patients With Monomicrobial vs Polymicrobial Acinetobacter baumannii-calcoaceticus Complex Infections Treated With Sulbactam-Durlobactam or Colistin: A Subset Analysis From a Phase 3 Clinical Trial
Source: Open Forum Infect Dis. 2024 Mar 20;11(4):ofae140. doi: 10.1093/ofid/ofae140 (PMC11002948; doi:10.1093/ofid/ofae140)
Supplement: ofae140_Supplementary_Data [file ofae140_supplementary_data.docx]

**Supplementary Materials**

**Comparison of clinical outcomes for patients with monomicrobial vs polymicrobial *Acinetobacter baumannii-calcoaceticus* complex infections treated with sulbactam-durlobactam or colistin: a subset analysis from a phase 3 clinical trial**

Sarah M. McLeod^1*^, Alita A. Miller^1,2^, Khurram Rana^1^, David Altarac^1^, Samir H. Moussa and Adam B. Shapiro^1^

^1^Entasis Therapeutics Inc., an affiliate of Innoviva Specialty Therapeutics, Inc.,

35 Gatehouse Drive, Waltham, MA 02451

^2^Author’s present affiliation: Arrepath, Inc., 303A College Rd East, Princeton, NJ 08540

*to whom correspondence should be addressed:

Sarah M. McLeod

Innoviva Specialty Therapeutics, Inc., an affiliate of Entasis Therapeutics Inc.

35 Gatehouse Drive

Waltham, MA, USA

[Sarah.McLeod@istx.com](mailto:Sarah.McLeod@istx.com)

**Table S1.** Phase 3 clinical outcomes and co-infecting pathogens for CRABC m-MITT patients with ABC polymicrobial infections treated with SUL-DUR/imipenem-cilastatin

| De-Identified Patient ID | Day 28 Survival (day of death) | Clinical outcome at Test of Cure | Baseline Gram-negative co-infection  (imipenem susceptibility) | Imipenem susceptibility restored by durlobactam in vitro? |
| --- | --- | --- | --- | --- |
| SUD-P1 | A | C | *P. mirabilis* (NS) | N |
|  |  |  | *K. pneumoniae* (NS) | Y |
| SUD-P2 | A | C | *E. coli* (S) | NA |
| SUD-P3 | A | F | *K. pneumoniae* (S) | NA |
| SUD-P4 | A | F | *E. coli* (S) | NA |
| SUD-P5 | A | C | None: *S. aureus* | NA |
| SUD-P6 | A | F | *K. pneumoniae* (S) | NA |
| SUD-P7 | D (19) | C | *S. maltophilia* (NS) | N |
| SUD-P8 | D (3) | F | *E. coli* (S) | NA |
| SUD-P9 | A | C | *P. aeruginosa* (S) | NA |
|  |  |  | *E. coli* (S) | NA |
| SUD-P10 | A | F | *A. xylosoxidans* (S) | NA |
| SUD-P11 | A | F | *P. mirabilis* (NS) | N |
| SUD-P12 | A | C | *P. aeruginosa* (S) | NA |
| SUD-P13 | A | C | *P. aeruginosa* (S) | NA |
| SUD-P14 | A | C | *K. aerogenes* (S) | NA |
|  |  |  | *P. aeruginosa* (NS), | NT |
|  |  |  | *P. mirabilis* (NS) | N |
| SUD-P15 | A | C | *S. maltophilia* (NS) | NT |
| SUD-P16 | A | I | *P. aeruginosa* (NS) | Y |
|  |  |  | *K. pneumoniae* (NS) | Y |
| SUD-P17 | A | C | *K. pneumoniae* (S) | NA |
| SUD-P18 | A | C | *K. pneumoniae* (NS) | N |
| SUD-P19 | **D (17)** | F | *K. pneumoniae* (NS) | Y |
| SUD-P20 | A | C | *K. pneumoniae* (NS) | Y |
| SUD-P21 | A | C | *K. pneumoniae* (NS) | Y |
|  |  |  | *P. aeruginosa* (NS) | Y |
| SUD-P22 | A | C | *K. pneumoniae* (NS) | Y |
| SUD-P23 | A | C | *K. pneumoniae* (NS) | Y |
| SUD-P24 | A | C | *K. pneumoniae* (NS) | Y |
| SUD-P25 | D (5) | F | *K. pneumoniae* (NS) | N |
| SUD-P26 | **D (20)** | F | *S. maltophilia* (NS) | N |
|  |  |  | *P. aeruginosa* (S) | NA |
| SUD-P27 | A | F | *K. oxytoca* (S) | NA |
| SUD-P28 | D (6) | F | *S. marcescens (S)* | NA |
| SUD-P29 | A | C | *K. pneumoniae* (NS) | Y |
| SUD-P30 | A | C | *K. pneumoniae* (NS) | Y |
| SUD-P31 | A | C | None: Coag-neg *Staph* | NA |
| SUD-P32 | D (28) | C | *P. mirabilis* (NS) | NT |
|  |  |  | *K. pneumoniae* (NS) | NT |

A, alive; D, dead; C, cure; F, failure; Y, yes; N, no; NA, not applicable; NT, not tested (isolate was not available for testing) Bold font, gray shading: death related to index infection. *Enrolled in Part B

(S), imipenem-susceptible (MIC ≤1 μg/mL for *Enterobacterales* and MIC ≤2 μg/mL for non-fermenters); (NS), imipenem-non-susceptible

**Table S2.** Phase 3 clinical outcomes and co-infecting Gram-negative pathogens for CRABC m-MITT patients with polymicrobial infections treated with colistin /imipenem-cilastatin (Part A)*

| De-Identified Patient ID | Day 28 Survival (day of death) | Clinical outcome at Test of Cure | Baseline Gram-negative co-infection  (imipenem susceptibility) | Gram-negative co-infection susceptible to colistin in vitro? |
| --- | --- | --- | --- | --- |
| COL-P1 | A | C | *P. aeruginosa* (NS) | Y |
| **COL-P2** | **D (14)** | **F** | ***K. pneumoniae* (NS)** | **Y** |
| COL-P3 | A | C | *K. pneumoniae* (NS) | N |
| COL-P4 | A | F | *P. aeruginosa* (NS) | Y |
|  |  |  | *A. xylosoxidans* (S) | Y |
| COL-P5 | A | C | *P. aeruginosa* (NS) | Y |
| COL-P6 | A | C | *E. coli* (S) | Y |
| COL-P7 | A | C | *S. marcescens* (S) | N |
| COL-P8 | A | C | *K. pneumoniae* (S) | Y |
| COL-P9 | A | F | *K. pneumoniae* (NS) | Y |
| COL-P10 | D (7) | F | *K. oxytoca* (S) | Y |
| **COL-P11** | **D (21)** | **F** | ***K. pneumoniae* (S)** | **Y** |
|  |  |  | ***P. mirabilis* (NS)** | **N** |
| COL-P12 | A | C | *P. aeruginosa* (NS) | Y |
| COL-P13 | A | C | *P. aeruginosa* (S) | Y |
|  |  |  | *S. marcescens* (S) | N |
| COL-P14 | A | C | *E. coli* (S) | Y |
|  |  |  | *K. pneumoniae* (NS) | Y |
| COL-P15 | A | F | *K. pneumoniae* (NS) | Y |
| COL-P16 | A | F | *K. pneumoniae* (NS) | Y |
| **COL-P17** | **D (27)** | **F** | ***K. pneumoniae* (NS)** | **Y** |
|  |  |  | ***P. mirabilis* (S)** | **N** |
| COL-P18 | A | C | *S. maltophilia* (NS) | Y |

*excludes a single patient documented as having a polymicrobial ABC infection with *H. influenzae* which was subsequently classified as a colonizing organism

A, alive; D, dead; C, cure; F, failure; Y, yes; N, no; NA, not applicable; NT, not tested (isolate was not available for testing)

Bold font, gray shading: death related to index infection.

(S), imipenem-susceptible (MIC≤1 µg/mL for *Enterobacterales* and MIC≤2 µg/mL for non-fermenters); (NS), imipenem-non-susceptible

**Table S3.** Carbapenem susceptibility in the presence or absence of durlobactam (DUR) or SUL-DUR of all available co-infecting imipenem-non-susceptible Gram-negative pathogens from patients treated with SUL-DUR in the Phase 3 trial

| **Patient ID**  **(de-identified)** | **Organism^1^** | **MIC (µg/mL) (susceptibility criteria^2^)** | | | | | | | | | |
| --- | --- | --- | --- | --- | --- | --- | --- | --- | --- | --- | --- |
|  |  | **DUR** | **SUL-DUR** | **IPM** | **IPM-SUL** | **IPM-DUR** | **IPM-SUL-DUR** | **MEM** | **MEM-SUL** | **MEM-DUR** | **MEM-SUL-DUR** |
| ***Enterobacterales***^3^ | | | | | | | | | | | |
| SUD-P1 | *Klebsiella pneumoniae*†† | 8 | 2 | 32 (R) | 64 (R) | 2 (I) | 2 (I) | >64 (R) | >64 (R) | 2 (I) | 2 (I) |
| SUD-P16 | *Klebsiella pneumoniae*†† | 8 | 2 | >64 (R) | >64 (R) | 2 (I) | 2 (I) | >64 (R) | 64 (R) | 2 (I) | 2 (I) |
| SUD-P18 | *Klebsiella pneumoniae*† | 4 | 8 | >64 (R) | >64 (R) | 16 (R) | 16 (R) | >64 (R) | >64 (R) | 16 (R) | 4 (R) |
| SUD-P19 | *Klebsiella pneumoniae* | 4 | 2 | 8 (R) | 8 (R) | 1 (S) | 1 (S) | 16 (R) | 32 (R) | 1 (S) | 1 (S) |
| SUD-P20 | *Klebsiella pneumoniae* | 1 | 0.5 | 4 (R) | 8 (R) | 1 (S) | 2 (I) | 16 (R) | 8 (R) | 0.25 (S) | 0.5 (S) |
| SUD-P21 | *Klebsiella pneumoniae* | 2 | 1 | 4 (R) | 4 (R) | 1 (S) | 0.5 (S) | 8 (R) | 8 (R) | 1 (S) | 1 (S) |
| SUD-P22 | *Klebsiella pneumoniae* | 4 | 2 | 8 (R) | 8 (R) | 1 (S) | 1 (S) | 32 (R) | 32 (R) | 1 (S) | 1 (S) |
| SUD-P23 | *Klebsiella pneumoniae* | 2 | 1 | 16 (R) | 4 (R) | 1 (S) | 1 (S) | 32 (R) | 32 (R) | 1 (S) | 1 (S) |
| SUD-P24 | *Klebsiella pneumoniae* | 4 | 4 | 8 (R) | 8 (R) | 1 (S) | 1 (S) | 16 (R) | 32 (R) | 1 (S) | 1 (S) |
| SUD-P25 | *Klebsiella pneumoniae*† | >64 | 4 | 64 (R) | 32 (R) | 16 (R) | 4 (R) | >64 (R) | 32 (R) | 16 (R) | 4 (R) |
| SUD-P29* | *Klebsiella pneumoniae* | 4 | 2 | 16 (R) | 8 (R) | 1 (S) | 1 (S) | 64 (R) | 32 (R) | 1 (S) | 1 (S) |
| SUD-P30* | *Klebsiella pneumoniae* | 4 | 2 | 4 (R) | 4 (R) | 0.5 (S) | 0.5 (S) | 32 (R) | 16 (R) | 1 (S) | 1 (S) |
| SUD-P1 | *Proteus mirabilis*†† | 8 | 8 | 2 (R) | 2 (R) | 2 (I) | 2 (I) | 0.06 (S) | 0.06 (S) | 0.06 (S) | 0.06 (S) |
| SUD-P11 | *Proteus mirabilis*† | 32 | 16 | 8 (R) | 4 (R) | 8 (R) | 8 (R) | 0.25 (S) | 0.25 (S) | 0.25 (S) | 0.25 (S) |
| SUD-P14 | *Proteus mirabilis*†† | 8 | 4 | 2 (I) | 1 (S) | 2 (I) | 2 (I) | 0.06 (S) | 0.03 (S) | 0.06 (S) | 0.06 (S) |
| **Non-fermenting Gram negative organisms^4^** | | | | | | | | | | | |
| SUD-P14 | *Pseudomonas aeruginosa* | 64 | 64 | 16 (R) | 16 (R) | 1 (S) | 2 (S) | 4 (I) | 4 (I) | 2 (S) | 4 (I) |
| SUD-P16 | *Pseudomonas aeruginosa* | >64 | >64 | 16 (R) | 16 (R) | 2 (S) | 2 (S) | 16 (R) | 16 (R) | 8 (R) | 8 (R) |
| SUD-P21 | *Pseudomonas aeruginosa* | >64 | >64 | 16 (R) | 8 (R) | 2 (S) | 2 (S) | 16 (R) | 16 (R) | 16 (R) | 8 (R) |
| SUD-P26 | *Stenotrophomonas maltophilia*† | 8 | 8 | >64 (R) | >64 (R) | 8 (R) | 8 (R) | >64 (R) | 64 (R) | 8 (R) | 8 (R) |
| SUD-P7 | *Stenotrophomonas maltophilia*† | 16 | 16 | >64 (R) | >64 (R) | 8 (R) | 16 (R) | >64 (R) | 64 (R) | 8 (R) | 8 (R) |

DUR, durlobactam; IPM, imipenem; MEM, meropenem; SUL, sulbactam.

^1^ All organisms had a sulbactam MIC ≥64 µg/mL.

^2^ Susceptibility interpretive criteria: (S) susceptible; (I) intermediate; (R) resistant.

^3^ For *Enterobacterales*, carbapenem-susceptible = MIC ≤1 µg/mL. The following combinations were titrated in two-fold dilutions: sulbactam-durlobactam, carbapenem-sulbactam and carbapenem-durlobactam in 1:1 ratios and carbapenem-sulbactam-durlobactam in 1:1:1 ratios.

^4^ For non-fermenting organisms, carbapenem-susceptible = MIC ≤2 µg/mL. Imipenem or meropenem was titrated in two-fold dilutions alone or in a 1:1 ratio with sulbactam. Durlobactam was added as a fixed concentration of 4 µg/mL.

^5^ Whole genome sequencing indicates that SUD-P18 encodes for β-lactamases TEM-1, SHV-11, CTX-M-15, OXA-1, OXA-9, OXA-48 and NDM-1 as well as a OmpK35 frameshift mutation at A59.

^6.^ Whole genome sequencing indicates that SUD-25 encodes for β-lactamases TEM-1, SHV-11, CTX-M-15, OXA-1, and OX-48 as well as frameshift mutations in OmpK35 at T254 and in OmpK38 at P130.

†Organisms that remained imipenem-resistant in the presence of durlobactam.

††Organisms that demonstrated intermediate imipenem susceptibility in the presence of durlobactam.

*Part B patient.
